# Disenrollment from general practitioners among chronic patients: a register-based longitudinal study of Norwegian claims data

**DOI:** 10.1186/s12875-016-0571-3

**Published:** 2016-12-15

**Authors:** Anastasia Mokienko, Knut Reidar Wangen

**Affiliations:** Department of Health Management and Health Economics, University of Oslo, P.O. Box 1089, Blindern, Oslo 0318 Norway

**Keywords:** Chronic patients, Switching, Primary health care, Schizophrenia, Epilepsy, Diabetes type 1, Diabetes type 2, Asthma, Arthritis, Depression

## Abstract

**Background:**

Norwegian general practitioners (GPs) consult on a variety of conditions with a mix of patient types. Patients with chronic diseases benefit from appropriate continuity of care and generally visit their GPs more often than the average patient. Our aim was to study disenrollment patterns among patients with chronic diseases in Norway, because such patterns could indicate otherwise unobserved GP quality. For instance, higher quality GPs could have both a greater share of patients with chronic diseases and lower disenrollment rates.

**Methods:**

Data on 384,947 chronic patients and 3,974 GPs for the years 2009–2011 were obtained from national registers, including patient and GP characteristics, disenrollment data, and patient list composition. The birth cohorts from 1940 and 1970 (146,906 patients) were included for comparison. Patient and GP characteristics, comorbidity, and patient list composition were analyzed using descriptive statistics. Patients’ voluntary disenrollment was analyzed using logistic regression models.

**Results:**

The GPs’ proportion of patients with a given chronic disease varied more than expected when the allocation was purely random. The proportions of patients with different chronic diseases were positively correlated, partly due to comorbidity. Patients tended to have lower disenrollment rates from GPs who had higher shares of patients with the same chronic disease. Disenrollment rates were generally lower from GPs with higher shares of patients with arthritis or depression, and higher from GPs who had higher shares of patients with diabetes type 1 and schizophrenia. This was the same in the comparison group.

**Conclusion:**

Patients with a chronic disease appeared to prefer GPs who have higher shares of patients with the same disease. High shares of patients with some diseases were also negatively associated with disenrollment for all patient groups, while other diseases were positively associated. These findings may reflect the GPs’ general quality, but could alternatively result from the GPs’ specialization in particular diseases. The supportive findings for the comparison group make it more plausible that high shares of chronic patients could indicate GP quality.

## Background

The quality of care for people with chronic diseases often relies on appropriate primary care. Some such patients may need continuous, long-term follow-up and motivation in order to maintain a favorable lifestyle. Others, who experience a condition associated with social stigma, may need time to develop trust in their care providers. Early detection of the chronic disease and its subsequent routine monitoring is also very important to save patients from acute hospitalization and complications from the disease [1]. Comorbidity is a good reason for primary care providers to be better able to manage chronic diseases [2, 3].

Previous studies have found that long-term physician-patient relationships are beneficial for patients [4, 5] and that patients disenroll from their general practitioner (GP) when they are not satisfied with their GP-patient relationship [6–10]. Patients may also disenroll from their GP if they perceive insufficient quality of care. Accessibility factors, such as adequate time for consultations [11] and availability of appointments [12] are predictors of good quality. Booking intervals for consultations and duration of the consultations themselves are correlated with good management of chronic diseases; the effect was greater for patients with asthma than for those with diabetes or angina, possibly because primary care providers deal more with asthma than diabetes or angina [13].

When it comes to accessibility, earlier research shows that longer patient lists are associated with negative evaluations of accessibility and that the GP's age has a negative association with the evaluation of all aspects, except accessibility [14]. Longer patient lists are also associated with better illness detection [15], which may suggest that practices detecting a higher number of chronic conditions have greater demand from patients due to their systematic chronic disease management [15–18].

A strong connection between patient choice and higher quality of practice, as measured by studying the publicly available data on practice performance, has been reported [19]. A review study found that patients were weakly influenced by publicly available information about provider quality [20]. On the provider side, only hospitals seemed to improve quality as a response to quality indicators being made publicly available [21]. For GPs, patient shortage has been found to correlate with patient dissatisfaction, the GP’s communication skills, and other GP characteristics [22–24].

Interaction between chronically ill patients and their GPs has not been given specific attention in previous literature, but a previous study of obese patients may contain clues for generalizable results: reportedly, obese patients avoided physicians they perceived as sources of stigma and searched for providers who were “obese friendly” [25].

If patients switch between GPs until their demands are met, we would expect these patients to be disproportionally distributed across GPs. Similar trends could be expected if the GPs intentionally specialize, formally or not, in a given patient group. However, neither of these mechanisms has obvious implications for the provider choices made by other groups of patients. For example, a GP who is popular among patients with diabetes type 2 (DT2) may also be popular among patients with depression, whereas patients without chronic diseases may be indifferent to this GP’s motivational skills. Older patients and patients with chronic diseases have generally higher care continuity, whereas patients with lower care continuity are those living in rural areas, employed, with higher education, or with poorer mental health [26].

Our aim is to investigate patterns of chronic patient disenrollment. This type of study is required because there are no published indicators of GP quality, and therefore these indicators need to be identified through patient actions (such as disenrollment). Moreover, specialized patient choice patterns might suggest an extra argument for using more fee-for-service reimbursement or risk-adjusted capitation for GPs in order to compensate for varying expected workloads depending on their patient list composition. Primary care in Norway is publicly funded with a capitation and fee-for-service system, and patients have to consult their GPs in order to see a specialist. Each individual GP has a patient list and can decide the maximum number of patients that can be enrolled on their list. Patients can switch between available GPs up to three times a year, according to their own preference.

## Methods

### Data sources and study populations

This is a retrospective study using data from two national registers in Norway, administrated by the Norwegian Directorate of Health, from 2009–2011. Our GP data were obtained from the national register of regular GPs, which covers the entire GP population, and merged with patient data using the GPs’ IDs. Our patient data were based on claims data obtained from the KUHR registry (Kontroll og Utbetaling av HelseRefusjon), which covers the entire Norwegian patient population. This registry records claims data continuously but for our analysis, the sample period 2009–2011 was divided into six semiannual intervals. The individual level data included patient characteristics, their consumption of primary care, and the GP with which they were enrolled.

Two samples of patients were selected among patients who visited a GP at least once from 2009–2011. Most of our analysis is based on sample 1, which consisted of patients registered with one or more of the following seven diagnoses at least once during the period 2006–2011: DT1, DT2, asthma, arthritis, schizophrenia, depression, and epilepsy. These patient groups were chosen because they are known to vary substantially both in the number of patients in the population, and in the utilization of primary care services. For instance, patients with DT2 constitute almost 5% of the population and receive most of their health care from their GP, while patients with schizophrenia are fewer and receive more specialist care in a hospital setting.

Our analysis also included a comparison group, sample 2. This group consisted initially of the entire birth year cohorts from 1940 and 1970, but we excluded patients already included in sample 1. Obviously this selection yielded an age distribution different from that in sample 1, but the selection of one elderly and one younger birth year cohort should provide a good basis for comparison.

**Fig. 1:**
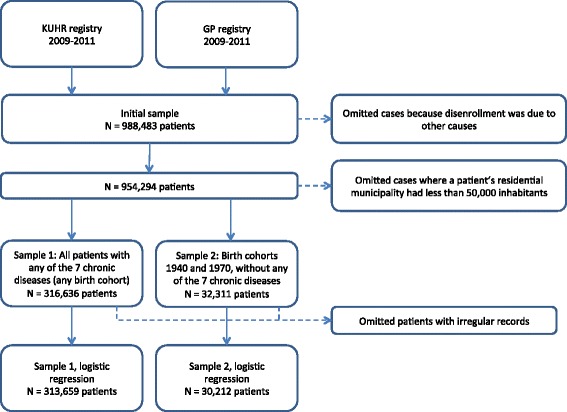
Flow diagram of sample selection

Initially, the two samples combined contained 988,483 patients (Fig. 1). We excluded 34,189 cases where the disenrollment was likely to be due to causes not relevant for our purpose; that is, when patients moved to another municipality, or when a GP moved, retired, or died. For the logistic regressions, we excluded patients living in municipalities with less than 50,000 inhabitants in order to focus on patients who could choose from several GPs. This left us with 316,636 patients in sample 1 and 32,311 patients in sample 2 (348,947 in total). Finally, we excluded patients with irregular medical records, mainly missing birth year or sex, yielding 313,659 patients in sample 1 and 30,212 patients in Sample 2 (343,871 in total).

### Measures

Our main outcome variable, ‘SwitchOut’, measured whether a patient disenrolled from a GP from one semi-annual period to the subsequent period. Definitions of independent variables are summarized in Table 1. Information about the GPs’ age, sex, specialization, and list length, and patients’ sex, birth year, and number of visits was obtained directly from the data registries. The variable ‘Pat_comorb’ was given the value 0 for patients in sample 2, while for each patient in sample 1 we counted the number of registered diseases (1–7) and subtracted 1 from this number. This yielded a variable with a range between 0 and 6. The variables ‘Diab2_share’ and ‘Epil_share’ measure a GP’s share of patients with the respective chronic disease, but with a slight adjustment: if shares were calculated straightforwardly, they could potentially be influenced by the health status of a single patient, because some chronic diseases are relatively rare and some GPs had fewer patients (shorter lists). To illustrate, consider a GP who has 100 patients, of which one has epilepsy. If we take the perspective of the GP, the share of patients with epilepsy is slightly above average (Table 1). However, this measure is of little relevance if we take the perspective of the patient with epilepsy: the GP has no other patients with epilepsy. To avoid interpretational ambiguity, we chose to take the patients’ perspective. For each patient-GP pair, we excluded the patient from the calculation of the GP’s share. Thus, the share variables mostly showed the variation between GPs but also some variation within a GP practice.

**Table 1:** Variable definitions and descriptive statistics on the patient level^1^

Variable	Definition	Sample 1 (*N* = 313,659)			Sample 2 (*N* = 30,212)		
		Median	Mean	St.dev	Median	Mean	St.dev
DT1_share	The share of a GP’s patients with diabetes type 1	0.005	0.007	0.006	0.005	0.006	0.005
DT2_share	The share of a GP’s patients with diabetes type 2	0.042	0.046	0.027	0.036	0.040	0.021
Arth_share	The share of a GP’s patients with arthritis	0.014	0.016	0.010	0.013	0.015	0.009
Asthm_share	The share of a GP’s patients with asthma	0.020	0.023	0.015	0.018	0.020	0.013
Depr_share	The share of a GP’s patients with depression	0.107	0.112	0.042	0.094	0.100	0.038
Schi_share	The share of a GP’s patients with schizophrenia	0.004	0.005	0.003	0.004	0.005	0.003
Epil_share	The share of a GP’s patients with epilepsy	0.008	0.009	0.004	0.008	0.008	0.004
ListLength	The number of patients on a GP’s list	1423	1444.0	367.8	1439	1453.4	367.8
Ln_ListLength	The natural logarithm of Listlength	7.261	7.240	0.277	7.272	7.248	0.270
GP_Age	The GP’s age	52	50.358	9.120	51	49.744	8.989
GP_Sex	=1 if the GP is male, =0 otherwise	1	0.706	0.455	1	0.673	0.469
GP_age · GP_Sex	The product of GP_Age and GP_Sex	48	36.473	24.718	45	34.266	25.016
GP_Specialist	=1 if the GP has a specialist degree in general medicine; =0 otherwise	1	0.707	0.455	1	0.702	0.457
Pat_Sex	=1 if the patient is male; =0 otherwise	0	0.426	0.494	0	0.494	0.500
Pat_BirthYear	The patient’s year of birth	1959	1958.6	19.1	1970	1961.5	13.5
Pat_Comorb	Sample 1: No. of chronic diseases minus one. Sample 2: Not defined	0	0.148	0.405	-		
Pat_Visits	The patient’s number of visits to primary care	3	4.662	5.268	1	2.227	3.369
Pat_Visits_win	Winsorized Pat_Visits at 99^th^ percentile (max = 23)	3	4.570	4.626	1	2.205	3.107
Pat_Visits_dum	=1 if Pat_Visit >23, =0 otherwise	0	0.10	0.98	0	0.002	0.047

^1^Municipalities over 50 000. First half of 2009

In order to avoid highly influential outliers, we transformed two variables. The distribution of GPs’ list length was skewed so we transformed the variable using the natural logarithm. The distribution of patients’ number of visits to primary care was also skewed, and for this variable, we winsorized the distribution at the 99^th^ percentile (23 visits per period) and included a dummy variable for observations that exceeded this limit.

### Statistical analyses

We inspected the data numerically and graphically at both the patient and GP levels. This included graphs intended to reveal whether the distribution of chronic patients seemed disproportionate across GPs. On the GP level, the mean proportion of patients with DT2 was 4.5% in the first half of 2009. If patients were allocated by pure chance, a randomly selected GP’s share of patients with DT2 would have the expected value of about 4.5%, and be approximately normally distributed for a sufficiently long patient list (>60 patients). For data at the GP level, we calculated Spearman’s correlation coefficients for the various GP-related variables, including the shares of patients with different diagnoses, the GP’s age and sex. We defined sub-samples of patients from sample 1 based on the seven chronic diseases. These sub-samples partly overlapped due to comorbidity. For each sub-sample, the shares of patients with 1 of the other six diseases were calculated.

We then used logistic regressions to model patients’ disenrollment from their GP. The modeling was performed for each patient category separately: on the sub-samples from sample 1, as defined above, and sample 2. Because the dependent variable (SwitchOut) was based on observations from two consecutive periods, we had up to five effective observations for each patient. For the independent variables, we used observations from the first five periods. The set of independent variables included those from Table 1, and an interaction term between GPs’ age and sex. We incorporated the longitudinal data structure by including patient-specific effects (intercepts) in the models. Patient-specific effects can account for unobserved factors, such as ethnicity or educational background, as long as these factors remain constant throughout the sample period. The models were estimated using xtlogit in Stata 13, under the standard assumptions that the patient-specific effects were normally distributed and did not correlate with the independent variables. Fixed effect models, which allow the patient-specific effects to be non-normally distributed or correlated with the independent variables, were also considered. However, in fixed effect models the time-invariant patient variables for sex and birth year would, by construction, be excluded from the estimations.

## Results

### Descriptive statistics

**Fig. 2:**
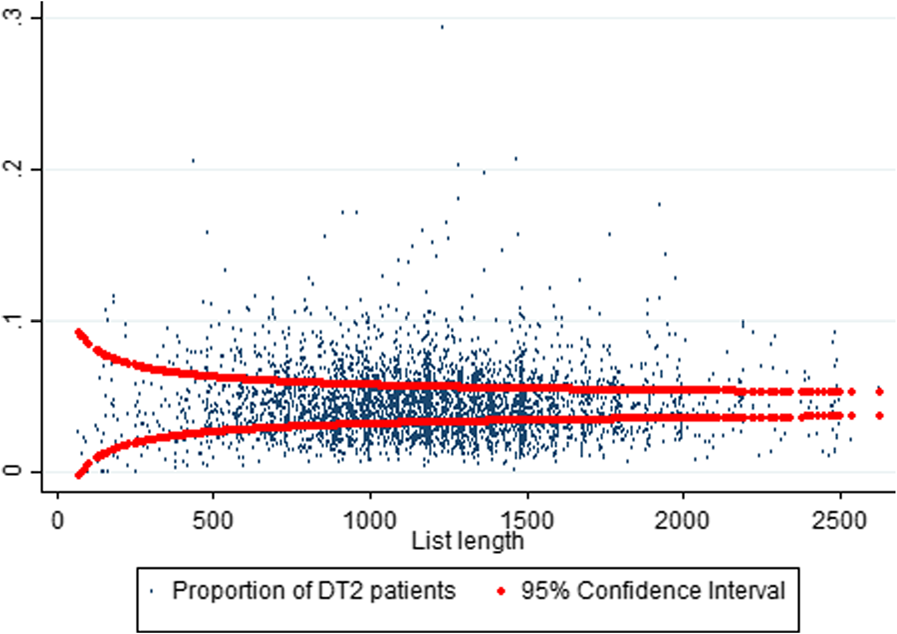
Scatter plot of GP proportion of DT2 patients and patient list length. Legend: Y-axis percent of DT2 patients, X-axis patient-list length. GP level, data for the first quarter of year 2009, *N* = 3,965, mean proportion of DT2 patients = 0.045, patient-list lengths of >60

According to Table 1 and Fig. 2, the proportion of patients with DT2 varied substantially among GPs. If these patients had been allocated purely by chance, about 95% of the proportions would lie between the red curves in Fig. 2, but this was not the case. In fact, only 46.5% of the proportions were positioned within the red curves. For the other diagnosis groups, the corresponding patient shares also seemed disproportionally distributed.

Overall, 4.5% of chronic patients disenrolled from their GP from one period to the next, but the share varied from 3.7% among patients with DT2 to 6.2% among patients with schizophrenia (Table 2). Among patients in sample 2, the share that disenrolled was 3.7%.

**Table 2:** Share of patients who voluntarily disenrolled from their GPs, between the 1^st^ and 2^nd^ halves of 2009.^1^

Sample	Sub set	N	%
Sample 1	Full sample	313,659	4.52
	DT1	11,292	4.99
	DT2	74,473	3.75
	Schizo	8,316	6.29
	Depr	186,415	5.00
	Arthr	27,157	4.00
	Asthm	37,110	4.16
	Epil	15,403	4.86
Sample 2	Full sample	30,212	3.76

^1^Municipalities over 50 000

Descriptive statistics for the independent variables used in the logistic regressions are reported in Table 1, separately for samples 1 and 2. Due to the sample selection procedure, the average GP characteristics differ somewhat from those obtained for the full GP population, where 66% were men, the average age was 48 years, and the average patient list length was 1200 (*N* = 3940).

The distribution of the variable ‘ListLength’ appeared continuous but was somewhat skewed to the right. The distribution of ‘Pat_visits’ was markedly right-skewed, and the distribution’s tail was rather scattered: for sample 2, the 75th, 95^th^, and 99th percentiles were 6, 14, and 23, respectively, but the maximum value was as high as 219.

Table 3 presents the sizes of the sub-samples defined for the seven chronic diseases. The most frequent of the diseases was depression (*N* = 488,686), while schizophrenia was the least frequent (*N* = 21,368). In the sub-sample of patients with depression (third column from the left), 1.3% also suffered from schizophrenia. Among patients with schizophrenia (rightmost column), 28.7% also suffered from depression. A substantial number of patients were recorded with both DT1 and DT2, likely due to registration errors or diagnostic uncertainty.

**Table 3:** Percent of patients with a chronic disease (column) that have another chronic disease (row)

	Arthritis	Asthma	Depression	DT2	DT1	Epilepsy	Schizophrenia
Arthritis		4.4	2.7	3.9	4.0	2.0	1.3
Asthma	6.1		4.5	6.7	5.8	3.6	6.0
Depression	14.5	17.6		13.5	15.2	15.8	28.7
Diabetes type 2	10.0	12.4	6.4		77.8	5.7	12.0
Diabetes type 1	1.6	1.7	1.1	12.0		1.3	1.9
Epilepsy	1.0	1.3	1.5	1.1	1.6		3.1
Schizophrenia	0.3	1.0	1.3	1.1	1.1	1.4	
N	90,095	124,776	488,686	232,383	35,887	46,145	21,368

^3^First half year of 2009. Patient level data. Sample 1 without restrictions (neither on municipality size, data irregularity or moving). N is the number of patients with the chronic disease

We calculated Spearman’s rank correlation coefficients for the GP proportion of patients with a given chronic disease and other patient proportions and GP characteristics, as shown in Table 4. The correlation coefficient of ‘Asthm_share’ and ‘DT1_share’ was 0.648, indicating that GPs with a high proportion of patients with asthma also tended to have a high proportion of patients with DT1. All variables related to the GPs’ proportions of patients were significantly different from zero. The proportion of patients with chronic diseases were all positively correlated, and negatively correlated with the proportion of other patients (‘Other_Share’). ‘Other_Share’ was negatively correlated with ‘GP_Age’ and ‘GP_Sex’, indicating that older GPs and male GPs tended to have fewer patients without our seven chronic diseases.

**Table 4:** GP characteristics. Spearman’s correlation coefficients with two-sided *p*-values.^2^

	Arth_ share	Asthm_ share	Depr_ share	DT1_ share	DT2_ share	Epil_ share	Schi_ share	Other_ share	GP_ age	GP_ sex	List Length
Asthm_share	**0.488**										
	0.000										
Depr_share	**0.195**	**0.264**									
	0.000	0.000									
DT1_share	**0.519**	**0.648**	**0.221**								
	0.000	0.000	0.000								
DT2_share	**0.232**	**0.310**	**0.121**	**0.332**							
	0.000	0.000	0.000	0.000							
Epil_share	**0.270**	**0.298**	**0.205**	**0.335**	**0.177**						
	0.000	0.000	0.000	0.000	0.000						
Schi_share	**0.045**	**0.175**	**0.227**	**0.135**	**0.183**	**0.162**					
	0.005	0.000	0.000	0.000	0.000	0.000					
Other_share	**−0.562**	**−0.683**	**−0.762**	**−0.712**	**−0.362**	**−0.406**	**−0.285**				
	0.000	0.000	0.000	0.000	0.000	0.000	0.000				
GP_Age	**0.203**	**0.137**	**0.064**	**0.213**	**−0.047**	**0.091**	−0.028	**−0.174**			
	0.000	0.000	0.000	0.000	0.003	0.000	0.077	0.000			
GP_Sex	**0.181**	**0.293**	**0.077**	**0.318**	**0.101**	**0.205**	**0.135**	**−0.265**	**0.249**		
	0.000	0.000	0.000	0.000	0.000	0.000	0.000	0.000	0.000		
ListLength	−0.020	**−0.069**	0.041	−0.032	**−0.145**	−0.040	−0.033	0.035	**0.166**	**0.172**	
	0.205	0.000	0.010	0.046	0.000	0.011	0.038	0.026	0.000	0.000	
GP_Specialist	0.008	0.017	0.030	0.037	**−0.133**	**0.067**	−0.003	−0.018	**0.365**	**0.098**	**0.226**
	0.618	0.275	0.063	0.020	0.000	0.000	0.860	0.250	0.000	0.000	0.000

^2^GP level data for first quarter of 2009, *N* = 3974. Correlation coefficients with two-sided *p*-values less than 1% are in boldface

### Logistic regression analysis

Table 5 shows the estimated parameters of the logistic regressions where ‘SwitchOut’ is the dependent variable, the independent variables are those listed in Table 1, and Sigma_u denotes the standard deviation of the patient-specific intercepts. The first seven columns show results based on sample 1 according to patient diagnosis group; the last column is based on sample 2. In logistic regressions, the coefficients can be used to compare the difference in log-odds ratios between groups, so that a patient sex coefficient of −0.188 (arthritis patients) represents the difference in log-odds ratios between male and female patients. The corresponding difference in odds ratios is obtained by taking the anti-log, exp(−0.188) = 0.829. The statistical inference for this type of model is based on large-sample theory and coefficient estimates are approximately normally distributed. Thus, to simplify the presentation, we do not report *p*-values as they can be derived from the estimated standard errors.

**Table 5:** Logistic regression for patients’ voluntary disenrollment from GPs, separate for patient groups.^4^Estimated parameters (standard errors)

	Arthritis	Asthma	Depression	Diabetes type 2	Diabetes type 1	Epilepsy	Schizophrenia	Others
Arth_share	**−15.032**	**−10.550**	**−16.792**	**−9.506**	**−16.905**	**−16.495**	**−20.113**	**−15.310**
	(1.611)	(1.597)	(0.815)	(1.194)	(3.116)	(2.836)	(3.925)	(2.185)
Asthm_share	**−4.381**	**−10.406**	**−2.117**	1.883	−1.624	−0.188	−3.895	0.093
	(1.598)	(1.309)	(0.636)	(0.934)	(2.494)	(2.262)	(2.922)	(1.799)
Depr_share	**−1.915**	**−2.343**	**−5.377**	**−2.781**	−0.484	**−2.029**	−1.095	−0.220
	(0.445)	(0.392)	(0.165)	(0.278)	(0.648)	(0.590)	(0.752)	(0.457)
DT2_share	−0.875	1.260	−0.534	**−4.117**	−0.499	−0.886	2.397	0.112
	(0.855)	(0.738)	(0.349)	(0.459)	(1.347)	(1.207)	(1.524)	(0.986)
DT1_share	**16.725**	**11.661**	**15.525**	**7.841**	**−20.177**	**15.491**	10.100	**15.962**
	(3.049)	(2.576)	(1.147)	(1.691)	(4.069)	(4.042)	(5.592)	(3.473)
Epil_share	9.578	**11.917**	**4.069**	4.048	−9.185	**−13.955**	−1.462	−0.165
	(4.637)	(3.910)	(1.695)	(2.815)	(6.681)	(5.882)	(7.709)	(4.754)
Schi_share	**23.551**	**28.298**	**37.453**	**39.029**	**21.821**	**39.502**	1.307	**29.586**
	(5.265)	(4.248)	(1.810)	(3.082)	(7.191)	(6.259)	(7.663)	(5.136)
Ln_ListLength	**−0.702**	**−0.631**	**−0.405**	**−0.658**	**−0.346**	**−0.489**	−0.205	**−0.623**
	(0.053)	(0.047)	(0.019)	(0.033)	(0.076)	(0.069)	(0.090)	(0.052)
GP_Age	**0.032**	**0.029**	**0.033**	**0.035**	**0.032**	**0.033**	**0.033**	**0.033**
	(0.003)	(0.003)	(0.001)	(0.002)	(0.005)	(0.004)	(0.006)	(0.003)
GP_Sex	−0.367	**−0.512**	**−0.202**	−0.234	−0.138	−0.108	−0.390	−0.317
	(0.189)	(0.166)	(0.065)	(0.118)	(0.265)	(0.235)	(0.306)	(0.175)
GP Age ^a^Sex	**0.010**	**0.013**	**0.006**	**0.007**	0.009	0.004	0.010	0.008
	(0.004)	(0.003)	(0.001)	(0.002)	(0.005)	(0.005)	(0.006)	(0.004)
GP_Specialist	**−1.148**	**−1.271**	**−1.145**	**−1.288**	**−1.119**	**−1.236**	**−1.189**	**−1.242**
	(0.035)	(0.030)	(0.012)	(0.021)	(0.050)	(0.044)	(0.056)	(0.033)
Pat_Sex	**−0.188**	**−0.090**	**−0.100**	**−0.082**	**−0.133**	0.015	**−0.163**	0.040
	(0.035)	(0.028)	(0.012)	(0.020)	(0.047)	(0.041)	(0.055)	(0.032)
Pat_BirthYear ^a^	**0.007**	**0.007**	**0.012**	**0.007**	**0.003**	**0.007**	**0.012**	**0.195**
	(0.001)	(0.001)	(0.000)	(0.001)	(0.001)	(0.001)	(0.002)	(0.036)
Pat_Comorb	**0.135**	**0.103**	**0.096**	**0.162**	**0.169**	**0.193**	**0.214**	
	(0.027)	(0.022)	(0.013)	(0.016)	(0.034)	(0.035)	(0.038)	
Pat_Visits_win	**0.042**	**0.046**	**0.049**	**0.041**	**0.035**	**0.049**	**0.046**	**0.057**
	(0.003)	(0.003)	(0.001)	(0.002)	(0.005)	(0.004)	(0.005)	(0.004)
Pat_Visits_dum	0.046	**−0.208**	**−0.270**	**−0.327**	−0.141	−0.157	−0.212	**−1.019**
	(0.116)	(0.087)	(0.046)	(0.083)	(0.158)	(0.134)	(0.143)	(0.333)
Cons	**−12.977**	**−13.367**	**−25.141**	**−13.052**	**−7.795**	**−14.111**	**−26.068**	−0.306
	(1.863)	(1.736)	(0.738)	(1.283)	(2.508)	(2.090)	(3.519)	(0.402)
Sigma_u	**0.718**	**0.784**	**0.773**	**0.747**	**0.755**	**0.809**	**0.922**	**0.662**
	(0.040)	(0.032)	(0.013)	(0.024)	(0.051)	(0.043)	(0.049)	(0.042)
No. obs	130,690	175,010	890,215	357,153	53,206	73,419	39,535	146,906
No. patients	27,157	37,110	186,415	74,473	11,292	15,403	8,316	30,212

^4^Dependent variable: ‘SwitchOut’. Only patients living in cities with more than 50,000 inhabitants were included. The seven left columns are from sample 1, the far right column is from sample 2. ^a^For ‘*Others’*, ‘*Pat_BirthYear’* was replaced with a dummy variable equal to 0 for patients born in 1940 and equal to 1 for patients born in 1970. Each patient was observed up to five times. Sigma_u denotes the estimated standard deviation of the random patient-specific constant terms. Stata 13, the xtlogit procedure, was used in the estimations. Estimates with two-sided *p*-values < 1% are in boldface

Some of the estimated effects of the patient share variables were relatively robust across patient groups. For ‘Arth_share’, all coefficients were significantly negative, implying that all patient groups tended to have lower disenrollment from GPs with relatively high shares of patients with arthritis. For ‘Asthm_share’ and ‘Depr_share’, all of the significant coefficients were also negative. In contrast, for ‘DT1_share’, ‘Epil_share’ and ‘Schi_share’, almost all significant effects were positive.

We can distinguish two main effects. First, the “own share effect,” namely, all patient groups tended to remain with GPs who had a high share of patients with the same diagnosis. Second, the “cross share effect,” where, for instance, a high share of DT1 patients increased the switch-out for patients with arthritis (meaning, patients with arthritis were more likely to switch-out if their GPs had more patients with DT1). The cross share effect was not generally symmetric as a high share of patients with arthritis reduced the switch-out for patients with DT1.

For all GP and patient characteristics, the significant coefficients had the same sign across all patient groups. Patients tended to switch less often from GPs who had long patient lists (‘Ln_ListLength’) or who were specialists in general medicine (‘GP_Specialist’). For older, female GPs, patients tended to switch out more often (‘GP_Age’). This effect was even stronger for male GPs, for which the full effect of age is obtained by adding the coefficients of ‘GP_age’ and the interaction between a GP’s age and sex (‘GP_Age*GP_Sex’).

Patients born more recently (i.e., lower ‘Pat_BirthYear’) or who had more comorbidities (‘Pat_Comorb’) tended to switch GPs more often. The 1% of patients who most frequently used primary care (i.e., ‘Pat_Visits_dum’ = 1) tended to switch less often than patients who had fewer visits. However, among the remaining 99% of patients, those with a higher number of primary care visits (‘Pat_visits_win’) tended to switch more often.

The patient-specific effects are assumed to be normally distributed, with a zero mean and an estimated standard deviation, Sigma_u. For patients with arthritis, the value of Sigma_u can be interpreted as the difference in log-odds between a patient who has a patient-specific intercept one standard deviation from the mean (0.718) and a patient with an intercept equal to the mean value (zero). This is about four times the numerical value of the coefficient for patient sex, and it corresponds to a difference in odds ratio equal to 2.050. In all patient groups, the estimated value for Sigma_u indicates that the unobserved patient characteristics have a comparably large influence on disenrollment.

## Discussion

Our data indicate that patients with chronic diseases are not allocated to GPs by chance alone (Fig. 2). One explanation could be that some GPs informally specialize, for example in DT2, and thus are able to establish and maintain a “stock” of such patients. In so doing, the patient comorbidity shown in Table 3 would imply a tendency for these GPs to also have relatively higher shares of patients with arthritis and asthma. Moreover, patients with chronic diseases tend to have comorbidities, contributing to their GPs having shares of patients with different diagnoses. This could partly explain why the proportions of chronic disease types are all positively correlated, as shown in Table 4.

The coefficients in Table 5 suggest that chronic patients disenroll less often from GPs who have a high share of patients with the same diagnosis; for instance, ‘Arth_share’ has a negative effect (−15.032) for patients with arthritis, and ‘Asthm_share’ has a negative effect (−10.406) for patients with asthma. Again, this may be the result of GPs informally specializing in certain types of patients with chronic diseases. It may also result from the GPs’ general qualities such as organizational skills, communication abilities, or empathic attitudes. It has been suggested that such patterns may result from patients’ negative interactions with healthcare providers, so that, for instance, obese patients search for “obese friendly” physicians [25]. Patients could also make use of informal conversations (word-of-mouth) with family, friends, or colleagues that recommend one GP or another, which seems to have a greater effect on the choice of GP than public information disclosure [20]. The relationship between the GP and patient could also be a factor in patient choice, since chronic patients spend more time in primary care and would change their GP if they were not satisfied [3, 4]. We can assume that GPs who have high numbers of patients with a particular disease might have a particular practice style, which also attracts these patients, but these mechanisms may be complex, for instance for patients with schizophrenia. In Table 5, the only exception from the general pattern is for patients with schizophrenia, for which the effect of ‘*Schi_share’* is insignificant. However, all other patient groups tend to disenroll more from GPs with high shares of patients with schizophrenia, potentially suggesting that these GPs are less popular in general, and this may perhaps counter the “own share effect” among patients with schizophrenia.

We find that all or most patient groups tend to disenroll less from GPs who have high shares of patients with arthritis, depression, and asthma. We assume that this disenrollment pattern happens due to qualities of GPs that attract most patients, such as good communication and care coordination skills. For chronic patients who are intensive users of primary care it is important to find a GP that fits their needs, so they might change until they find the right match. Patients in the comparison group have, per se, no obvious reason to prefer GPs who specialize in any chronic disease, but it is likely they have preferences regarding GP qualities. Thus, our finding that in some cases the preferences of the comparison group and of the patients with chronic diseases align suggests that GPs’ shares of chronic patients reveals information about these GPs’ general qualities.

A puzzling finding is that all or most patient groups tend to disenroll more from GPs who have high shares of patients with DT1 and schizophrenia. According to Norwegian guidelines, these two patient groups’ follow-up happens in secondary care, in contrast to our other patient groups. Patients who receive follow-up in secondary care could perhaps be more indifferent to which GP they visit for other acute illnesses. If so, they may be satisfied with GPs who have a practice style favoring patients who can be treated expediently over patients who need long-term follow-up. With this interpretation, the high disenrollment among patients with schizophrenia (Table 2) can be interpreted not necessarily as a search for a GP who is well-suited for handling issues related to schizophrenia but perhaps as an expression of other, shorter-term considerations.

GP specialization in general medicine has a negative relationship with disenrollment, suggesting that patients prefer to stay with specialized GPs. List length also has a negative relationship with disenrollment for all patient groups, except for patients with schizophrenia. Previous studies have found that non-chronic patients stay with GPs with shorter patient lists, meaning that they value accessibility [10–12], in contrast to chronic patients who value long patient lists, which is associated with higher disease detection [13]. GP’s age is positively related with disenrollment for all patient groups, suggesting that patients in general may prefer younger GPs. This effect of age is supported by earlier findings [12]. For patients with arthritis, asthma, depression or DT2, this tendency is stronger for male than female GPs, perhaps because there are fewer women among older GPs than among younger GPs. In most patient groups, disenrollment was not significantly associated with GP sex, except patients with asthma and depression, who tend to less often disenroll from male GPs.

In all groups of patients with chronic disease, disenrollment increased with the number of comorbidities. This is consistent with the discussion above, given that management of patients with comorbidities is challenging for primary care providers [27]. Our selection of patient groups was not, however, designed to investigate the effect of comorbidities in particular. Future studies should consider including other diagnoses, such as cardiovascular disease and cancer. A higher number of visits to primary care also tended to increase disenrollment, but the negative coefficients for the dummy variable, identifying patients who had more than 23 visits in a six month period, may indicate that the relationship between disenrollment and the number of visits is not linear. Younger patients generally disenroll more often and, except for patients with epilepsy and other patients (sample 2), male patients disenroll less often.

This study has three main imitations: first, although the majority of the numerical data seemed reliable, we found that as many as 77.8% of patients with DT1 were also registered as having DT2. Such “double diabetes” cases are not uncommon [28, 29], but it is likely that most of the cases in our data are due to diagnostic uncertainty or registration errors. This may affect both the results related to the share of patients with diabetes (‘DT1_share’ and ‘DT2_share’), and the results for sub-samples defined for patients with DT1 and DT2. Second, our data did not include potentially relevant patient variables such as cultural background, native language, income, educational background, or marital status. Disease severity and proper control of symptoms could also influence disenrollment behavior. To an extent, our random effect logistic regressions can account for time-invariant patient variables, but future studies should consider including more variables in order to assess their influence. Additional information about the GPs, such as cultural background, length of time in practice, and professional interests would also have been of interest. Third, the age distribution differs between our selected comparison group, sample 2, and our main sample of interest, sample 1. Sample 2’s age distribution also differs from the age distribution across all groups in the full population without our specified chronic diseases. This means that the estimates for sample 2 in Tables 2 and 5 are likely to be biased, if interpreted as estimates for the full population. We believe that the qualitative aspects of these results would not be very different in the full population, but this is of course a conjecture. Future register-based studies should consider obtaining a comparison group with similar age distribution as the sample of main interest, for instance by drawing patients randomly from the entire population.

The data sets used in our logistic regressions were restricted with respect to municipality size. In smaller municipalities, patient options for disenrollment will be more limited by the fact that there are fewer local GPs to choose from. It is likely that including patients irrespective of municipality size would yield estimated effects less pronounced than those reported here – that is, compared to the full population, our result are likely to be biased away from zero. We also excluded observations where observed disenrollment seemed to be due to causes other than patients’ preferences for GPs. Patients and GPs who move, or GPs who retire or die, are likely to have demographic characteristics (e.g., age) that differ systematically from the distributions in the full patient and GP populations. It is more difficult to predict how including these observations would have influenced our results, but it would at least have complicated the interpretations.

## Conclusions

The following conclusions can be drawn from our findings: 1) patients with chronic diseases are not allocated to GPs only by chance; 2) chronic patients that use primary care intensively disenroll less often from GPs who have a high share of patients with the same diagnosis; and 3) most patient groups tend to remain with GPs with a greater share of arthritis, asthma, and depression patients, which can indicate better quality care for these and other patient groups. These conclusions are distinct from the findings in the literature.

To investigate this further, more objective quality measurements should be obtained, such as adherence to treatment guidelines, surveillance of treatment outcomes for chronic patients, and user satisfaction in general. If objective quality differences are found, further assessments could be warranted, for instance, whether the current reimbursement system has an appropriate balance between capitation and fee-for service, or whether capitation should be risk-adjusted based on shares of patient types.

## Abbreviations

DT1: Type 1 diabetes
DT2: Type 2 diabetes
GP: General practitioner
KUHR: Control and payment of reimbursements to health service providers (Kontroll og Utbetaling av HelseRefusjon)
